# The impact of patient involvement in research: a case study of the planning, conduct and dissemination of a clinical, controlled trial

**DOI:** 10.1186/s40900-020-00214-5

**Published:** 2020-07-19

**Authors:** Pernille Christiansen Skovlund, Berit Kjærside Nielsen, Henriette Vind Thaysen, Henrik Schmidt, Arnstein Finset, Kristian Ahm Hansen, Kirsten Lomborg

**Affiliations:** 1 Experimental Clinical Oncology, Department of Oncology, Aarhus University Hospital, Palle Juul-Jensens Boulevard 99, 8200 Aarhus N, Denmark; 2 Department of Oncology, Aarhus University Hospital, Palle Juul-Jensens Boulevard 99, 8200 Aarhus N, Denmark; 3 The Research Centre for Patient Involvement, Aarhus University & the Central Region, Palle Juul-Jensens Boulevard 99, 8200 Aarhus N, Denmark; 4 DEFACTUM, Social & Health Services and Labour Market, Central Denmark Region, Olof Palmes Allé 15, 8200 Aarhus N, Denmark; 5 Department of Surgery, Aarhus University Hospital, Palle Juul Jensens Boulevard 99, 8200 Aarhus N, Denmark; 6 Department of Behavioural Sciences in Medicine, Institute of Basic Medical Sciences, University of Oslo, Domus Medica, Gaustad, Sognsvannsveien 9, 0372 Oslo, Norway; 7 Department of Clinical Medicine, Faculty of Health, Aarhus University, Incuba Skejby, building 2, Palle Juul-Jensens Boulevard 82, 8200 Aarhus N, Denmark; 8 Steno Diabetes Center Copenhagen, Niels Steensens Vej 2, 2850 Gentofte, Denmark

**Keywords:** Patient and public involvement, Co-creation, Analysis, Patient-reported outcomes

## Abstract

**Background:**

The interest in patient and public involvement (PPI) in health research is increasing. However, the experience and knowledge of PPI throughout the entire research process and especially in the analysis are limited. We explored ways to embrace the perspectives of patients in a research process, and the impact and challenges our collaboration has had on patients, researchers, and the research outcomes.

**Methods:**

This is an explorative single case study of a Danish, clinical, controlled intervention trial and a nested intervention fidelity study included herein. Five patient representatives with metastatic melanoma were part of designing, undertaking and disseminating the trial where the effect of using patient-reported outcome (PRO)-measures as a dialogue tool in the patient-physician consultation was tested. In the fidelity study, audio-recorded consultations were analyzed after training in the Verona Coding Definitions of Emotional Sequences (VR-CoDES). Results were jointly disseminated at an international scientific conference. The outcomes, impact, and challenges were explored through a workshop.

**Results:**

In the design phase, we selected PRO-measures and validated the dialogue tool. The information sheet was adjusted according to the patients’ suggestions. The analysis of the fidelity study showed that patients and researchers had a high consensus on the coding of emotional cues and concerns. The patients contributed with a new vocabulary and perspective on the dialogue, and they validated the results. PPI caused considerations related to emotional (sadness/sorrow and existential thoughts), administrative (e.g. arranging meetings, balancing work and small talk) and intellectual (e.g. avoiding information harm, continuing activities despite the death of patients) investments. A limitation of the study was the lack of use of a solid evaluation tool to determine the impact of PPI.

**Conclusion:**

PPI throughout the process and co-creation in the analysis was feasible and beneficial. The case is unique in the degree of workable details, sustainability, and transparency. Moreover, the co-creation provides ideas of ways to operationalize PPI. An evaluation workshop revealed considerations about emotional, administrative and intellectual investments – best described as tacit, yet important ‘work’. This knowledge and experience can be applied to other studies where patients are partners in the research.

**Trial registration:**

ClinicalTrials.gov ID: NCT03163433, registration date: 8th May 2017.

## Plain English summary

Despite an increasing interest in patient and public involvement (PPI) in health research, the experience and knowledge of PPI throughout the entire research process and in particular in doing the analysis are limited. We involved five patients in designing, undertaking and disseminating a clinical trial. In the trial, the effect of using specific questionnaires as a dialogue tool in the consultation between clinicians and patients with metastatic melanoma was measured. In the trial’s design phase, patients were involved in selecting questionnaires in making the information sheet. Undertaking the trial, patients and researchers analyzed audiotaped consultations separately after receiving training together. We did this to see if the questionnaires were used as requested and to see if patients and researchers analyzed the consultations alike. We found agreement on the analysis, but the patients added a different perspective and new words to explain the dialogue than the researchers. A researcher and a patient presented the case of the PPI collaboration jointly at an international conference. At an evaluation workshop, some considerations emerged - like arranging meetings, avoiding information harm for patients and continuing activities despite the death of patients. These are emotional, administrative and intellectual investments and best described as tacit, yet important ‘work’. Patients had considerations about the sharing of sensitive topics, offering themselves in discussions, and how their work had contributed to the trial. This knowledge and experience can be applied to other studies where patients are partners in the research process.

## Background

Patient and public involvement (PPI) in research refers to the inclusion and activation of patients or public laypeople as partners in the various stages of the research process, or as *“research being carried out ‘****with****’ or ‘****by****’ members of the public rather than ‘****to****’, ‘****about****’ or ‘****for****’ them*” [1]. There is increasing interest in PPI in health research [2, 3]. In particular, in North America, the UK, and Canada, PPI in health research is well established and supported by organizations and frameworks. These include Patient-Centered Outcomes Research Institute (PCORI) in the United States, INVOLVE in the UK, and SPOR in Canada [2–4]. In Denmark, PPI is still in its early stages, and hence not yet as well developed and incorporated in clinical research produced in Denmark as in the US, UK or Canada. Consequently, Danish researchers look at INVOLVE and others for guidance. However, several Danish patient associations as well as research funds in general have begun to require a statement of PPI in their call for research proposals. The Danish Cancer Society is one of the first patient organizations in Denmark to make specific requirements for PPI in applications for research grants; one study that received funding has since published an article on the subject [5].

Being a pioneer in the area of PPI, INVOLVE distinguishes between three PPI approaches: consultation, collaboration and patient-led research [1]. ‘Consultation’ refers to patients being asked about their opinion on specific areas chosen by the researchers; in ‘collaboration’ or ‘co-production’ patients and researchers work together in a more or less equal relationship, and in ‘patient-led research’ patients deliver and manage research themselves, with assistance from researchers [6]. Similarly, Health Canada divides PPI into five stages: inform or educate, gather information, discuss, engage and partner [7]. Methods and approaches for conducting PPI are applied with great variation and cover a broad range of areas, such as interviews, focus groups and surveys [1, 8].

Several different frameworks support PPI in research [9]. Especially three prevailing arguments supporting PPI exits: 1) patients have a right to have an input to research on their condition; 2) patients hold a lived-experience perspective that can improve the efficiency and value of research by increasing relevance, improving recruitment and retention rates, and improving dissemination of findings beyond academic audience; and 3) PPI increases the accountability and transparency of research and may be an effective way of attracting resources [9].

PPI in health research is believed to benefit quality, relevance and clinical results [1, 10], and can influence the choice of research topics and direction of research, project design and methods, recruitment and data collection, analysis, and dissemination [1, 11, 12]. Public involvement may also positively affect the researchers by making them reevaluate their thoughts on the research process [4, 11]. Studies show that, in clinical trials, patient involvement can lead to improvements in design, selection of relevant outcome measures [8] and effective recruitment of trial participants [13].

PPI in research can take place at every phase of the research project. INVOLVE has illustrated a research cycle in seven phases: identifying and prioritizing, commissioning, designing and managing, undertaking, disseminating, implementing, and evaluating impact [1]. Studies where PPI have been applied have mostly focused on early phases of the research cycle (e.g., prioritizing the research question, developing research design and planning a recruitment strategy) and on the dissemination of results [3, 8, 12]. However, since patients are rarely involved in the data collection or data analysis, little is known about how to engage patients in conducting a study [14]. Lack of PPI in this part of the research process, in which data is analyzed, may lead to the loss of valuable perspectives in the interpretation and validation of findings [6]. In addition, experience and knowledge of expanding PPI to several research phases or throughout the entire research process is limited [3, 8, 15].

An evaluation of the impact of PPI is warranted, not least in studies investigating the use of patient reported outcomes (PRO) in cancer consultations [3, 16, 17]. Furthermore, critical reflections, including both desirable and undesirable experiences of PPI, are lacking [3], and the reporting of PPI activities is often poor or omitted [12]. Unraveling some of the complexity of PPI impact can be done via the Public Involvement Impact Assessment Framework (PiiAF) [18] and the Guidance for Reporting Involvement of Patients and the Public (GRIPP) checklists (short and long format) [19, 20]. Each provide a comprehensive tool to improve the reporting of patient and public involvement in research.

**Fig. 1:**
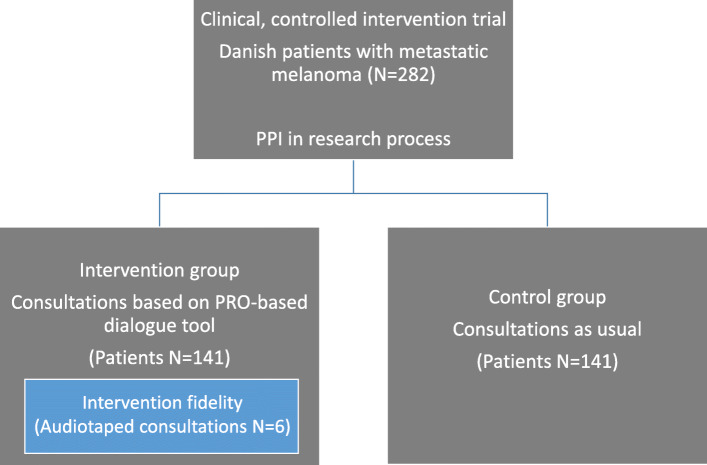
Overview of the clinical intervention trial

Better descriptions are required on how, where and when engagement has been implemented, in order to improve understanding of PPI’s potential beneficial impacts [12] and to document how the activities in particular phases of the PPI process are done. In this article, we discuss the impact of engaging patients and researchers as partners in all phases of a clinical research project and, in particular, in performing the analysis. Furthermore, we examine: a) ways in which the perspectives of patients with metastatic melanoma can be embraced in health research, b) the impact of this collaboration on patients, researchers, and the research outcomes, and finally, c) the possible challenges and considerations that need to be addressed. The clinical research project is a Danish, clinical, controlled, intervention trial where patient representatives became patient research partners (PRPs) as members of a patient advisory board. We investigated the effect of using PRO-measures as a dialogue-based tool in consultations between clinicians and patients with metastatic melanoma. The intervention group completed PRO-measures before each consultation, and answers were fed back to their electronic medical records. Clinicians could then prepare for the consultation and focus the dialogue on what the patients found most important to discuss. The control group did not use PRO during consultations. Nested in the intervention, a pragmatic intervention fidelity study was included, see Fig. 1. Intervention fidelity refers to the degree to which an intervention is delivered as intended, and is a strategy to monitor and enhance the consistency and integrity of the intervention [21]. The intervention took place at a large cancer clinic at a university hospital while patients in the control group were recruited from another two Danish university hospitals.

## Methods

This is an explorative single case study, where the research cycle illustrated by INVOLVE served as a framework for engaging patients in every phase of the research project: “Feedback in the consultation – a dialogue-based tool in personalized care planning using systematic patient involvement”. During the research process, the level of involvement grew from consultant to collaboration and co-creation as described by INVOLVE [1]. We especially focused on the co-creation of the analysis, since this is one of the less explored phases of the research cycle. Thus, our study is “an empirical experiment in its natural setting without control over actual behavioural events by the investigator” [22, 23]. It thus embraces multiple sources of data and provides a highly detailed, contextualized analysis of “an instance in action” [22]. The GRIPP checklist (long format) was used as a guidance of reporting PPI in this paper, see additional file 1.

### The case

The case (Table 1) represented the involvement of patients with metastatic melanoma throughout the research process. In the phases of “Identifying and prioritizing” and “Commissioning”, patients from the Danish Cancer Society were involved in prioritizing research topics and choosing which projects to fund. These phases are not described further in this case, because those patients did not engage in the advisory board of the clinical trial. Furthermore, the phases of “Implementing” and “Evaluating” are still at the planning stage, and will only briefly be covered.

**Table 1:** The case of PPI in the research project

**The advisory board**	
Before recruitment of PRPs, the six researchers (a cancer consultant, two clinical professors (one specialized in patient involvement), a professor in patient involvement, a health psychologist, a clinical nurse specialist, and a nurse Ph.D. student (principal investigator (PI)) agreed that the researchers had scientific responsibility, but the PRPs had the responsibility to bring forward the lived experience. Accordingly, the aim of the advisory board was to ensure quality, enable completion of the trial, and keep the intervention meaningful to patients and health care professionals. For the recruitment of PRPs, an open invitation was announced on the Facebook group site of the Danish melanoma network. The network has about 180 paying members and 890 members in their Facebook group. We assumed that patients who joined such an organization were likely to have the required skills and resources to contribute to an advisory board. We asked for patients who had experiences of living with metastatic melanoma and the treatment hereof within the last 5 years, and who believed they could express viewpoints and experiences to the researchers. Furthermore, patients should be able to communicate via email and to attend 6–12 meetings over a project period of 3 years. Four patients applied for the three positions. They were approached by email and telephone in order to align expectations. The three included patients represented a diverse range of experiences of living with metastatic cancer (from 2 months to 6 years) but a narrow age range (62, 65, and 65) and gender (only males). Due to the death of two patients within the first 1.5 years, two new patients were recruited through the same Facebook group. Two females (aged 52 and 49 with 1.5 years and 4 months of living with metastatic melanoma, respectively) joined the group after individual telephone contact, followed up by project information. One of the new members had a progression of her disease shortly after the telephone contact and never joined the following meetings.	
**The overall framework of activities**	
All meetings took place at the university hospital from February 2016 to November 2019 and had predefined agendas. In order to accommodate the participation of patients who were in the labour market, the meetings were mainly organized after normal working hours and lasted for 3 h. At least one meal was served at each meeting and a refund of PRPs’ travel expenses was offered.	
**Activities in the design and management phase**	
In this phase, information about the PRPs’ motivations for and expectations of joining the advisory board were gathered, and a clear division of tasks and responsibilities was discussed. The main activities were selecting of PRO measures and composing the patient information sheet. All meetings were planned and managed by the PI with a mixed level of engagement, alternating between consulting and collaboration in decision-making.	
**Activities in the analysis during the undertaking phase**	
We established an ad hoc sub-group consisting of four researchers and two PRPs, all interested in a qualitative analysis of how the PRO dialogue tool was used in practice. The two RPRs were the only ones left in the advisory board (due to death and progression of disease). The aim of the ad hoc sub-group was two-fold: 1) to determine pragmatic intervention fidelity by monitoring if PRO as a dialogue tool in the clinical trial was applied as intended, and 2) to investigate if patients would identify elements of perceived significance in the consultations, which the researchers alone would not notice. Audio-recorded consultations were collected and coded according to Verona Coding Definitions of Emotional Sequences (VR-CoDES) [24, 25]. The VR-CoDES can be used to analyze emotional communication in medical consultations. The focus is on patients’ emotional expressions, whether they are explicit (direct concerns) or indirect (cues), and the immediate response by the clinician [24–26]. Co-inventor and author of this coding system, Professor Arnstein Finset trained the ad hoc sub-group prior to the analysis and assisted the coding process. Applying the VR-CoDES rendered possible an analysis of PRO as a facilitator for further disclosure of emotions. In this phase, the level of engagement was co-creation, where all ad-hoc sub-group members contributed equally. Since the PRPs needed to meet the same requirements as the researchers, they were remunerated for their work on the analysis. To capture and evaluate the impact of PPI on PRPs, researchers and outcome, the ad hoc sub-group held an interactive evaluation workshop a couple of months after the coding in the analysis phase was finalized.	
**Activities in the dissemination phase**	
One patient representative and PI participated in an international conference with a poster and panel discussion on PPI and contributed to the preparation and execution of this paper. It is planned that the PRPs will be involved in the dissemination of the results of the clinical project.	

### Data material and analysis

The data material included multiple sources of data gathered from: email correspondences between PRPs and the PI; sticky notes about expectations and possible personal contributions; coding schemes; records of the proceedings, written by the PI and subsequently approved by all members; discussion-notes; and audio recordings from the evaluation workshop.

The coding and analysis of the pragmatic intervention fidelity study were made individually by all participants of the ad hoc sub-group, in accordance with the VR-CoDES, and were compared with Professor Finset’s codes of the same consultations. Consensus about the codes was reached after a discussion about our individual codes.

The analysis of the impact of PPI on PRPs, researchers and research outcome was qualitatively explored through all the above-mentioned sources of data, particularly from consensus on records of the proceedings and the workshop. At the workshop, fundamentals of the patient-researchers’ collaboration, challenges and outcomes of PPI were explored through dialogues moderated by an independent researcher who was not involved in the project, in order to illuminate any potential blind spots within the group.

## Results

### The involved patient research partners

Collaboration between patients with metastatic cancer and researchers has its immediate advances such as honest input from patients, inspiring, dynamic discussions, and new professional relationships. However, it also has its challenges. The patients’ cancer might progress, and they might get too ill to participate. Out of the five patients involved in this trial, only two patients are currently active partners in the advisory board. Patient characteristics of all PRPs at the time of joining the project can be seen in Table 2.

**Table 2:** Characteristics of patient representatives

	Patient 1	Patient 2	Patient 3	Patient 4	Patient 5
Gender	Male	Male	Male	Female	Female
Age at project initiation	65	65	62	52	49
Work-life at project initiation	Self-employed, former director	Director in own company	Retired, receiving a special pension	Nurse	Social educator
Cohabiting	Married	Married	Married	Unknown	Married
Diagnosed with metastatic melanoma	6 years	5 years	2 months	1½ years	4 months
Current treatment or place in course of the disease	First-line treatment for 3 years. Routine appointments every 4th week	Currently off treatment after receiving 3 different lines of anti-cancer treatment. Routine appointments every 3rd month	First-line treatment for two months. Routine appointments every 3rd week	Second-line treatment for the last 8 months. Routine appointments every 4th week	First-line treatment for four months. Routine appointments every 3rd week
Motivation for joining the advisory group	● Curiosity	● Gain insights into how patients can contribute to making a better consultation	● Learn more about my disease	● Help eliminate chaotic feelings for other	● Help other
	● Use own experiences				● Use the disease to do something constructively
	● Payback for treatment and care		● Spread knowledge about the disease		
		● Use own experiences		● Help others in the same situations	
		● Gain knowledge about the use of PRO	● Gain insider-knowledge		● Take control over own disease
				● Help myself	
Self-assessed potential contributions	● Experience and theoretical knowledge of questionnaires	● Personal experiences with PRO	● How it feels to be a patient	● Experiences with cancer treatment	● Experiences with transitions between departments
		● Ability to help draw up PRO with high value for patients	● Knowledge about psychological issues connected to metastatic melanoma	● I never give up	
					● Experiences with not getting answers to your questions
	● Experience of own illness				
		● Refine PRO			
	● Used to deliver and contribute to groups				● Experiences with the disease, treatment, and follow-ups
			● Knowledge about the process of being diagnosed with cancer		
Previous experience of involvement in research projects as a patient representative	None	None	None	None	None

It is apparent from Table 2 that the PRPs had various different motivations for joining the advisory board, and that none joined with a negative attitude or due to mistrust in the health system. They wanted to use their lived experiences in a meaningful way to help others and give something back to the system that had helped them.

### PPI activities in the research process

**Fig. 2:**
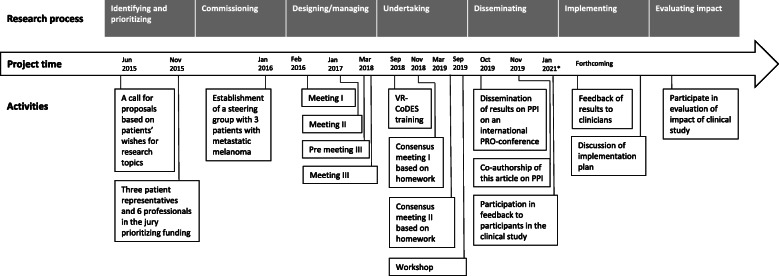
Overview of PPI-activities and time flow

In Fig. 2, an overview of the meetings in the advisory group and the ad-hoc sub-group is displayed in connection with the timeline and the research process.

In the “Designing and managing” phase, three meetings were held to choose PRO-measures for the dialogue tool and for the research project, to compose the patient information sheet, and to decide on a relevant design for the pragmatic intervention fidelity study. An additional meeting between the PI and the new PRPs took place to inform the latter about the advisory board and our common, future work within the group. The “Undertaking” phase comprised a joint training day for both researchers and PRPs, two consensus meetings, at which the codes were discussed, based on individual code-work done as homework, and an evaluation workshop. In the “Dissemination” phase, several meetings were held between the PI and one patient representative. This patient had been awarded a PRP scholarship to attend a conference together with the PI to present the work and thoughts on PPI. This PRP also engaged in the writing of the present article. During the research process, the PPI approach grew from consultation to co-creation. A detailed description of the activities in each of the phases, together with the levels of involvement, and the output and outcome, can be found in Table 3. PPI in the Undertaking phase will be further explored below, as an example of patient engagement in data analysis.

**Table 3:** Detailed overview of research PPI-activities, output and outcome

Aim	Activities	Output	Outcome
**Design and managing phase**			
Meeting I. Length: 3 h, level of involvement: Consultation to Collaboration			
- To clarify expectations and motivation for participation	- Individual sticky notes and subsequent sharing of expectations and motivations	- List of expectations and motivations for joining the advisory board	- The researchers used the contributions from the patient representatives to select PRO-measures for the dialogue tool. The chosen PRO-measures included health-related quality of life plus anxiety and depression. The final decision about PRO-measures was validated by the patient representatives in e-mail correspondence
- To inform patient representatives about the clinical study		- Assurance that the selected PRO-measures are sufficient to prioritize what is important for patients	
	- Dias show about the overall study purpose and design		
- To choose PRO-measures for a dialogue tool		- Recommendation of PRO-measures on psychological issues in the dialogue tool	
	- Exposition of three PRO-measures selected prior to the meeting by the researchers as potential PRO-measures for the dialogue tool		
			- The relatives were considered, but to include them would be too wide-ranging. In the final dialogue tool, patients were encouraged to complete PRO together with relatives
- To choose PRO-measures for research		- Suggestion of PRO-measures for relatives because relatives play a major role for many patients in managing their disease	
			- The participants were asked to state their three most important issues to discuss
		- Recommendation that completion of PRO-measures must be followed by a conversation	
			- Questionnaires designed according to an estimated max time consumption of 15 mins.
	- Group work:		
	Completing the PRO-measures		- An IT solution for completion at home and at hospital
		- Endorsement of an open-ended question on the most important issues to discuss in the consultation	
	Discussing PRO-measures’ suitability to address patients’ concerns		- “self-efficacy” and “health-related quality of life” were among domains in the final outcome “battery”, but the potential outcome was difficult to separate from the desired or important outcome
		- Advice on length of the questionnaire: 15 mins for each completion is acceptable	
	Selecting PRO-measures		
	Discussing the length of the total dialogue tool	- Advice on completion: preferably at home in a comfortable environment but possible at hospital	
	Discussing completion time		
		- Suggestion about outcome measures: “self-efficacy” and “health-related quality of life”	
	- Exposition and discussion of relevant domains of PRO-measures for research		
Meeting II. Length: 3 h, level of involvement: Consultation to Collaboration			
- To complete a user-friendly patient information sheet	- Dias show about the decisions made since the last meeting	- Suggestion for a new title because the original was perceived as too long and not catchy	- The new title was used
			- All suggested rewritings were implemented
- To brainstorm on a training manual for clinicians using PRO in the dialogue			- Information on a meeting for participants when results are available was included in the information sheet
	- Group work:	- Sentence by sentence rewriting to shorten the text	
	Is the information sheet adequate?		
		- Rewriting of words that were incomprehensible in order to make it user-friendly	- The final version was sent out by e-mail and approved in the advisory board
All members received two draft versions before the meeting; one for the intervention group and one for the control group			
	Are the language and grammar easy to understand?		- Due to lack of time, the patient representatives were not further involved in the development of a training manual
		- Advice to arrange and inform about a meeting to feed back results to participants as a “pay-back” for participation in the clinical trial	
	- Short presentation of ideas for a training manual on PRO for physicians		
	- Discussion about these ideas (due to lack of time, this was a brief discussion (5 min))	- No specific ideas for the training manual besides the ideas presented by PI, but the involvement of clinicians was highlighted as an important next step	
Pre-meeting III. Length: 1 h, level of involvement: Not applicable			
- To introduce two new patient representatives	- Face-to-face meeting with one new patient representative	- Willingness to contribute as a patient representative	- Both new patient representatives were included in the advisory board
Inform about the clinical project, PPI, and responsibilities			
	- Telephone meeting with another patient representative		
Meeting III. Length: 2 h, level of involvement: Consultation to Collaboration			
- To welcome the new patient representatives	- Information about different methods for the intervention fidelity (e.g., interview, observation, audio recording)	- A joint decision to use audiotaped recordings of consultations	- Establishment of an ad-hoc sub group
- To inform about methods of monitoring, documenting and analyzing the way PRO is used in the consultation		- Willingness to engage as co-creators in the analysis process	- Contacted the founder of the coding system professor A. Finset in order to set up training and work schedule
	- Information on estimated process and time schedule for collecting and analyzing data	○ Spend the additional time needed to engage in this work	- Signed contract about confidentiality and salary
- To select an intervention fidelity study design		○ Obtain training in a specific coding system	
	- Discussion and decision about method and level of PPI		
		○ Code separately	
		○ Meet for discussions	
**Undertaking phase**			
Training^a^. Length: 7 h, level of involvement: Collaboration			
- To learn how to code audiotaped consultations according to VR-CoDES	- Training in theory and method of the coding system	- The patient representatives engaged in the entire training session	- Knowledge to apply VR-CoDES to other transcripts on audiotaped consultations
- To decide how to map the use of PRO in the audiotaped consultations	- Practical exercise in coding exercise transcriptions	- Joint discussion and decision about how to code the use of PRO	- Practical experience of using the VR- CoDES
	- Supervision by professor A. Finset		- Agreement of a plan for the mapping of the use of PRO in the consultation (six questions to address the use of PRO)
	- Discussion on monitoring the use of PRO in consultations		
Homework I^a^. Length: 3–4 h, level of involvement: Co-creation			
- To apply the VR-CoDES to transcripts of audiotaped consultations	- Individual coding (part I) of three consultations with audiotaped length of 11–26 min. Each (7–13 A4 pages each)	- Individual training and familiarity with the VR-CoDES	- The underlying basis for collaboration on the analysis
- To code the use of PRO		- Coding of all three audiotaped consultations	
		- Focus on PRO in the consultations	
Consensus meeting I^a^. Length: 3 h, level of involvement: Co-creation			
- To compare and discuss the individual application of VR-CoDES	- Comparing our codes with each other’s and with the codes produced by A. Finset	- Expressions of being emotionally touched by the confrontations with fellow patients’ hard consultations	- Consensus on how to code
			○ We all agree on how to code a patient’s emotional concern or hint of concern, but the character of the hint or the response by the physician is more complicated to code, leading to varied views on why a patient hints various concerns and why physicians respond as they do
- To compare and discuss the use of PRO	Typical agreements and disagreements	- Emphasizing the importance of the patient-physician relationship in regard to the communication flow	- Consensus on the use of PRO
			○ The three most important issues to discuss are used as a starting point of the dialogue
	Discussion about consensus		
		- Emphasizing the importance of the patient’s position in the course of a disease for the character of hints	○ Difficult to tell if PRO symptoms and function are used
	- Comparing our answers to the six questions to address the use of PRO		
			○ Mainly, the physician initiates the dialogue
		- Arguments on how a physician response can depend on how experienced a physician is	
		- Joint mapping of how PRO was referred to, initiated and used	
Homework II^a^. Length: 4–5 h, level of involvement: Co-creation			
- To apply the VR-CoDES to transcripts of audiotaped consultations	- Individual coding (II) of three consultations with an audiotaped length of 14–60 min each (9–24 A4 pages each)	- Individual training and familiarity with the VR-CoDES	- The underlying basis for collaboration on the analysis
- To code the use of PRO		- Additional focus on the use of PRO in the consultations	
		- Coding of all three audiotaped consultations	
Consensus meeting II^a^. Length: 3 h, level of involvement: Co-creation			
- To compare and discuss the individual application of VR-CoDES	- Comparing our codes to each other’s and to the codes produced by A. Finset	- One patient representative told about hearing information about prognosis that she had tried to avoid in relation to her own disease	- None of the patient representatives regret having been involved in the co-production
- To compare and discuss the use of PRO	Any typical agreements and disagreements		- The ad-hoc sub-group is more aligned in the codes than at last consensus meeting
		- One patient representative highlighted that a repeated question from a patient to a physician may not indicate an underlying concern, but might indicate an unacceptable physician response	- A new vocabulary and perspective to talk about the dialogue based on PRO in the consultation
- To discuss the impact of co-creation so far and going forward	- Comparing our answers to the six questions to address the use of PRO		- Validation of cues and concerns found (by patient representatives)
			- The dialogue tool was used as intended in all the audiotaped consultations
			- The analysis revealed that the open-ended questions were the starting point of the dialogue, but we were unable to tell if the validated PRO-measurements were used during the consultations
		- Recognition of the concerns and the questions in the audiotaped consultations	
		- Joint mapping of how PRO was referred to, initiated and used	
Workshop on evaluation of the impact of PPI^a^. Length: 2½ hours, level of involvement: Co-creation			
- To work out challenges in doing PPI in our case	- Discussions about engaging in this research project based on an interactive presentation software	- Guiding points for patient representatives and researchers in doing PPI, e.g.:	- Co-creation was feasible in the case
- To determine the impact of PPI on the patient representatives, the researchers and the outcome			- PPI was beneficial throughout the research process in order to incorporate the perspectives of patients with metastatic melanoma
		○ Clarify responsibility and expectations explicitly	- The validation of output and outcome assisted in a recognition of own contributions
	- Gathering advice for other patients and researchers who engage in PPI in research		
		○ Be aware of information harm	
		○ Create room for trust and respect to unfold	
	- Validation of contributions and outcomes		
		○ Be aware of expenses (time and money)	
		- Validation of the outputs and outcomes presented by PI. A few more were added	
**Disseminating phase**			
Meeting I^b^. Length: 2½ hours, level of involvement: Co-creation			
- To plan participation at an international conference on quality of life research (patient research partner scholarship obtained)	- Discussing different invitations to participate in session and panel discussions about patient engagement in research	- Joint decision to participate at one session as panelists and in one session as an active audience member	- Feedback to the session organizers about our decisions about participation
			- Joint performances to demonstrate and display our cooperation in the research process
- To discuss the first draft of a poster made by PI	- Discussing the content, layout, and presentation of the poster	- Poster	- Poster finalized by PI included the input from the patient
		○ Layout is agreed upon as introduced by PI	
		○ Content is agreed upon, but with the idea to outline considerations about PPI in research from both the researchers’ and patients’ perspectives	
Tele-conference with organizers and panelists^b^. Length: 1 h, level of involvement: Not applicable			
- To receive an introduction about the session in which we were to become panelists	- Meeting the organizers and other panelists	- Initial preparation as panelists	- The initial preparation for the joint performance
	- Discussing the format and questions for the panelists		
Meeting II^b^. Length: 2 h, level of involvement: Collaboration			
- To finalize the preparation for the conference	- Discussing the questions for the panelists	- Final preparation for the conference and our performances	- Confidence in our performance
	- Brainstorming answers for panel questions		- Check on practicalities, such as travel documents and the conference programme
	- Discussing travel and conference programme		
Participation at ISOQOL conference 2019^b^. Length: 4 days, level of involvement: Collaboration			
- To present our work on PPI together	- Poster presentation on PPI in research	- Numerous responses to our presentations	- International acclaim for our work on PPI in cancer care
- To network with researchers and stakeholders interested in patient engagement	- Panel discussion on patient engagement in research		- International network
	- Informal dinner with Special Interest Group for Engagement		
	- Participating in sessions about PPI		
Meeting III^b^. Length: 2 h, level of involvement: Collaboration			
- To go over the paper based on a thorough, individual perusal	- Discussion of results and discussion paragraph	- One patient representative participated in publishing the present article	- Agreement between all authors
- To obtain consensus on the content	- Writing of layman summary	- Layman summary written by patient representative and PI	
	- Review and approval of the article		
Meeting IIII^b^. Future planning, level of involvement: co-creation			
		- Feedback of results to clinicians is to be planned in detail	
		- Feedback of results to participants is to be planned in detail	

^a^ Participants: Ad hoc sub-group within the advisory group

^b^ Participants: PI and a patient representative

### PPI in data analysis of the intervention fidelity study

The outputs by the ad hoc sub-group and the impact these had on the outcome, indicates that co-production in the analysis was feasible. The PRPs were in position to participate in a one-day training workshop and they subsequently individually applied the trained coding system to audio-recorded consultations of fellow patients. In interactive group discussions at two consensus meetings, we found that PRPs and researchers reached a high level of consensus about the clinical use of PRO and that PRO-data was used as intended in the audiotaped consultations. Most often, the physicians initiated the dialogue based on the PRO-data. The starting point of every dialogue were open-ended questions about the most important issues that the patient wanted to discuss. However, it was impossible to determine if PRO-data – such as patient answers regarding symptoms and functions – were used during the consultation. Additionally, we established a high consensus around the VR-CoDES. We generally coded unanimously and found no evidence of PRPs identifying emotional expressions that the researchers did not identify. The patients deemed the coded emotional cues and concerns to be identifiable and valid. This may indicate a robust coding system. However, PRPs contributed with new perspectives on how the physician-patient relationship and the patients’ position in their disease trajectories, determine the way emotional expressions and responses are articulated in the consultations. Furthermore, the PRP’ knowledge led the ad hoc sub-group to interpret words slightly differently to the system. For example, in the VR-CoDES, a patient’ use of swear words suggests an underlying unpleasant emotion, but in one of the audiotaped consultations, a patient cursed throughout the entire consultation including in sequences that the ad hoc sub-group deemed free of emotions (such as a discussion about the weather). This lead the PRPs to suggest that swearing was this patient’s way of speaking in general instead of an emotional cue. The same applied in another consultation, where the PRPs suggested that a patient’s use of humor in a description of symptoms could be linked to a geographical origin of the patient instead of linked to an unspecific way to describe an underlying unpleasant emotion. The experience with patient coding confirms the impression that coding according to VR-CoDES rules always should take context into consideration, and that input from patients regarding contextual factors may increase the validity of coding. Moreover, the PRPs broadened the views on the link between physicians’ responses to emotional expressions and their professional experiences of similar patients. By doing so, they used their lived experiences to reveal an understanding of the patient-clinician relationship that had been hidden to the researchers.

### PPI-output, outcome, and impact

The above example shows how PPI impacted on how words and expressions were interpreted. The PRPs broadened our understanding of how emotional expressions are dependent on the patient-physician relationship and the disease trajectory. Besides these perspectives, the co-creation of the analysis also made sense to the PRPs. One of them said:

*With this task, I felt my involvement in this project could make a difference – I was really engaged in and committed to identifying emotional concerns that the researchers and the coding system overlooked.*

Even though the PRPs identified no additional emotional concerns, this statement indicated that they felt important throughout the process.

To capture the contribution of PPI on the research project, all decisions were written down in records of proceedings during the process. In addition, all participants in the evaluation workshop validated and extended the results. As illustrated in Table 3, contributions from PRPs were identified as outputs of PPI activities. For example, one output was the PRPs’ recommendation to include PRO-measures on psychological issues in the dialogue tool, and an endorsement of open-ended questions on the most important issues to discuss in the consultation. The PRPs deemed this to be extremely relevant. The output led to the selection of PRO-measures focusing on health-related quality of life, anxiety and depression, and included space for free text to fulfill the recommendations from the patients. Another output was the PRPs’ advice to arrange an information meeting to feedback results of the clinical trial to participants as a payback for their participation in the clinical trial. As a result, this output led to a notification in the written material report that all participants would be invited to an information meeting when the results of the clinical trial were available. These two examples of PRPs’ outputs and derived outcomes demonstrate new perspectives and the impact of PPI on the design and organization of the clinical trial.

The derived outputs and outcomes of PPI listed in Table 3 were all related to the research project and seen from a research perspective. However, in mapping the impact of PPI, the evaluation workshop revealed that outputs and outcomes did not make the same impact on everybody and that some outcomes of conducting PPI are not related to the trial study outcome but, rather, to the study process or the collaborating persons.

By coincidence, one of the PRPs received unwanted information on prognosis when listening to a fellow patient’s audiotaped consultation. This ethical dilemma was an outcome of co-creation that made an impact on all ad hoc sub-group members. We became concerned that other information could also cause harm (information harm). However, this had no impact on the clinical trial. Likewise, administrative elements, such as arranging meetings, determining salary and insurance and obtaining approval only impacted the researchers and had no impact on PRPs or the clinical project. The death of PRPs and the progression of the disease had an impact on both PRPs and researchers on a personal level. We were all affected and sad to realize that our fellow group members were no longer with us. In addition to the emotional and existential impact that the death of PRPs had on everybody, their deaths also had an impact on our common workload (introducing new members) and group dynamics (welcoming and getting to know new members), but none of these factors had an impact on the clinical project.

### Challenges of doing PPI

PPI is a time consuming, emotional, cognitive, and practical effort. At the workshop, it became clear that all members faced challenges with PPI, even though there was variation in the challenges. Table 4 contains some of our challenges and some of the considerations these challenges call for – from both the PRPs’ and the researchers’ perspectives. From the researchers’ perspective, deciding which PRPs, how many and where from needed consideration in the recruitment phase. The balance between leaving the PRPs with a feeling of too little PPI leading to no significant impact or leaving them feeling overwhelmed with work and responsibilities involved much deliberation by the PI. Likewise, much time and effort were spent on keeping everybody informed so that they could make joint decisions in a genuine collaboration. Furthermore, it was challenging when PRPs progressed in their disease and died during the research process. The need to convey sad news to fellow advisory board members, acknowledge and process feelings of identification with the deceased patients’ disease trajectory, while, at the same time, maintaining and continuing the project activities and recruiting and informing new patients, meant additional considerations, time and effort.

**Table 4:** Examples of challenges and considerations of doing PPI

Challenges of doing PPI in research	Examples of considerations from the researcher perspective	Examples of considerations from the PRPs’ perspective
Funding	- Where to apply and how to budget PPI	- None in this case
	- Changing the level of PPI requires additional funding	
	- Travel expenses and salary	
Recruitment of patient representatives	- Which patient representatives (gender, age, patients or relatives, pointed out and asked at the hospital or an open invitation through the patient association)	- Worries about living up to certain expectations must be put aside in order to sign up for engagement
		- Worries about asymmetric dialogues between academic people and layman must be put aside
	- Number of patient representatives (represent the entire patient group, balanced with the number of researchers)	
Level of PPI	- Consultation, collaboration, co-creation, user-led	- Openness about abilities and feelings of inadequacy
	- Change of level over time according to research question and request and abilities among patient representatives	
Administrative investment	- Money investment (costs of meetings, salary, and reimbursements for PRPs, teaching needed in the analysis, funding for participation in a conference)	- The costs PPI may have on everyday life (e.g. time spend, confrontations with hard feelings) must be acceptable
	- Time and place of meetings to accommodate wishes from both patient representatives and researchers	- Alignment of PPI-activities with relatives
	- Arranging meals and snacks to pay back to patient representatives and to maintain a cozy atmosphere	
	- Constant follow-up at meetings or by e-mail on how a task or homework has been received by the patient representatives	
	- Balance time between small talk and work. Both are essential when doing PPI	
	- Individual introduction to new members and encouragement to active and equal participation	
Intellectual investment	- Inclusion and discussion of all thoughts and ideas – even when these do not match each other	- Sharing of sensitive topics
		- Offering yourself in discussions
	- Respect and trust in each other in order to capture true experiences	- Willingness to be honest
	- Listen to, acknowledge and consider all comments	- Dealing with insights that might be difficult to separate from your own situation
	- Addressing and maintaining an agreed-upon division of tasks and responsibilities	
		- Worries about own contributions
	- Avoiding information harm	- Direct reference to PPI contributions by PI is the easiest way to recognize own impact as a patient representative
	- Concerns about work or responsibility overload for the patient representatives	
	- Concerns about conference participation (performance on panel presentation, understandable topics, welcoming atmosphere)	
Progression of disease or death of patient representatives	- Respect for a patient representative’s choice to cut down on activities or to stop completely	- Open dialogue
	- Open dialogue in the group	

Moving from an involvement level of consultation to collaboration and co-creation meant an even bigger time investment and higher workload. Finding out about salary, insurance, and funding for the PRPs was not easy. As PPI is rather new in Denmark, there are no widespread guidelines, and what might be regarded as “good practice” regarding honoraria in some countries may not be applicable to a Danish setting where we have a long tradition of voluntariness in clinical research. The PRPs did not request or even expect honoraria, but we wanted to acknowledge their part in the analysis as equally important to the researchers. This is exceptional in Denmark, and since we were without much precedence in Denmark, we looked to INVOLVE for inspiration and offered salary according to the payment guidelines made by INVOLVE [27]. In conducting the analysis, ethical concerns arose around giving approval to PRPs to listen to tough consultations where fellow patients disclosed their fears. There was a risk of harm from being exposed to too much or unwanted information. Finally, additional time, energy and resource investments included coordinating the dissemination of results, applying for additional patient representative scholarships, preparing for joint conference participation and covering travel costs incurred.

From the patient’s perspectives, signing up for engagement in a research process for the first time is challenging in itself, as it involves entering a new work arena with potentially asymmetric relations. There were fears of not being able to fit in, understand and contribute to the research. Additionally, the balance between spending time and effort on a research project versus spending time with family also required consideration and the need for adaptation within the family.

As seen in Table 4, many of the considerations were related to administrative and intellectual investments. These represented investment in a meaningful PPI collaboration, but they were difficult to document, because the work was best described as tacit ‘work’. Despite the character of this tacit ‘work’, we found it to be just as essential as respect, sincerity, and openness in harnessing PPI. Maintaining authentic and sustainable collaborations between PRPs and researchers depends on respect, equality, and trust, which call for time and effort from everybody involved. Strong collaborations are not built overnight, they need nurturing. This includes meetings, ongoing correspondence, and constant follow-ups to maintain contact during periods in which no meetings are held.

## Discussion

The case of PPI in the project “Feedback in the consultation – a dialogue-based tool in personalized care planning using systematic patient involvement” is an example of an authentic and sustainable operationalization of collaborations during the life cycle of a research project. Throughout the research process, the partnership was characterized by empathy and respect for each other, for our different backgrounds and contributions, and for our mutual project. The partnership lasted more than 3 years, despite the death and disease progression of some PRPs. To our knowledge, this is the first example of co-creation with metastatic cancer patients that report findings from such a long-lasting process.

### Balance of PPI investment versus gains

PPI in health research requires huge investments (e.g., time, financial resources, and emotional and intellectual effort), not only by research organizations and teams, but also by the patients who get involved [28]. The sustainability of PPI in a project depends largely on the balance between investments in PPI and the gains, in terms of better research (relevance, feasibility, trustworthiness, credibility, adherence to protocol, etc.). In line with previous studies [12, 29], we found that PRPs’ contributions helped improve various aspects of the intervention, such as designing the questionnaires according to an estimated, patient-stated max time consumption of 15 min, selecting patient-requested PRO-domains, and producing user-friendly information. Additionally, the PRPs validated the findings in the pragmatic intervention fidelity study and led the ad hoc sub-group to interpret some words differently to the VR-CoDES. This could indicate that coding of audiotaped consultations can or should be interpreted in the light of its context, and that PRPs could contribute in that process. However, the tacit ‘work’ described earlier and the amount of planning that goes into the conducting of a research project with PPI (as illustrated in Fig. 2 ) indicate a need for a careful attention on the balance between investment and gain. The fact that no formal economic assessments were performed during the case confounds the picture of the balance.

### PPI skills

Working collaboratively requires a range of skills, particularly various interpersonal skills and coordination skills – the latter which are required when handling the many administrative tasks displayed in Table 4. Interpersonal skills are necessary to establish and maintain a positive, conducive collaboration. Through the evaluation workshop, we found that respect, trust, honesty, and keenness towards PPI were fundamentals in a meaningful collaboration. This is in line with other studies [6, 30, 31]. Especially, when upgrading the level of PPI from consultation, which involves discrete, one-way interactions, to more relational approaches in co-creation, thought should be given to engaging in meaningful collaborations and establishing an atmosphere where sincere thoughts and feelings can be shared. This calls for a focus – beyond the project – on relationship building [32], such as allowing room for small talk and acknowledging and addressing well-intentioned, yet contradictory, ideas. Furthermore, it was found – in our case and previous studies – that the PI needed to frequently and clearly report back to PRPs about the ways in which their input was contributing to the study [11, 17].

### Co-creation in the analysis

Considering the small number of studies on PPI in the analysis, and the absence of methodologies for collaborative data analysis [6], our pragmatic intervention fidelity study provides a meaningful and transparent contribution to the important knowledge gap on PPI in the data analysis. The involvement of patients in the analysis of transcripts has previously been done in a study on patients’ experiences of stroke and depression [33]. Among other aims of PPI in that study, one was to see if patients would identify themes (touchpoints) that were similar or different to the researchers. Like in our case, the patients and researchers identified very similar touchpoints. This may indicate that even though involving patients in data analysis is feasible, the added contribution of lived experience is hard to determine. The authors of that study concluded that even a small sample of data was too ambitious in terms of time available, suggesting that when involving patients in analysis, the focus should be on conversation and guidance in the further analysis, rather than data [33]. In our case, the solid training of the ad hoc sub-group contributed to a clear process, which enabled a deeper understanding of emotional expressions in clinical consultations based on PRO – and especially of open-ended questions as a facilitator of patient-centered consultations. Based on the codes and the PRPs’ lived experiences, elements such as patient-clinician relationship, disease trajectory, and professional expertise were highlighted by PRPs as determining elements of how emotions were expressed and responded to during consultations. These perspectives added a valuable layer to our understanding of the patient-clinician dialogue and were not solely a starting point or guidance for the analysis. In fact, this indicates that precious knowledge can be lost if PRPs are not engaged in data analysis. This is supported by another study on PPI in the analysis of data on service users’ discussions of Cognitive Behavioural Therapy (CBT) for psychosis [14]. The authors concluded that multiple coding is an important method for understanding and exploring multiple perspectives on data and building team consensus. We therefore recommend that the challenges of PPI in the analysis (e.g., information harm) should not prevent engagement in co-creation, however it must be articulated from the beginning as a possible condition of doing PPI.

### Limitations

Our design was a single case study with a wide range of sources to inform our results and a high degree of detail. Generally, case studies are not perceived as strong evidence. There is nothing to which it can be compared, and we cannot rule out the possibility that the same results would have been found without PRPs.

Only five patients in total, and two patients in the analysis phase, participated as PRPs during the process. The fact that all PRPs had a higher-level educational status indicates that they possessed some level of methodological competencies in research, which they used. This could limit the transferability of our findings to other contexts where PRPs may have a lower level of education. Nevertheless, the small number of participants made possible a deeper relationship within the advisory board, thereby facilitating an open, honest and rich involvement.

Patients and clinicians in the pragmatic intervention fidelity study were aware of the audio recorder leading to a better compliance with the protocol than consultations not being recorded. Therefore, these consultations may not be representative of the actual use of PRO in the consultation. However, the audiotaped consultations could indicate an evaluation of clinicians’ adherence to and competence with the intervention, as well as the patients’ ability to understand how to use PRO and apply this in the consultation [34, 35]. The small number of audio recordings and the unblinded nature of the pragmatic intervention fidelity are considered limitations of the intervention, although they do not relate specifically to PPI.

Although a workshop had been proposed in an earlier study, to measure the impact of patient-engaged research [36], the robustness of such a workshop can be questioned, because no solid evaluation tool was used to determine the impact. At our workshop, the understanding of the impact was built through dialogues. The workshop revealed no potential negative impacts of PPI. Participants of the advisory board may have felt reluctant to speak critically about the case and PPI, given that they had developed it themselves and given that the evaluation was not anonymous.

### Strengths and future perspectives

The use of INVOLVE’s definition of PPI and their research cycle provided an applicable and beneficial framework for the demonstration of PPI activity across the research process [1, 11], which is a strength of the study. This article and, in particular, the high level of detail in Table 3 can be used as inspiration when doing PPI in future studies. Lessons can be learned from our study. For example, we would consider mitigating the potential negative impacts of patients dying, suffering information harm, or dropping out during the PPI work by allowing a greater number of PRPs in future studies in order for the PPI work not to become vulnerable, and maybe designing studies where patients intentionally enter as PRPs at different time points during the process dependent on the tasks. Most of all, the importance of designing studies allowing enough time to focus on emotional well-being at every meeting, offering an open and honest dialogue, and having a plan for debriefing from the outset can be important lessons learned from this case. This goes for researchers who plan PPI, PRPs who may demand these elements, and patient associations or other funds that finance the projects.

## Conclusion

Collaboration between researchers and PRPs throughout the research process was feasible and beneficial in this trial. Especially in the undertaking phase, the PRPs contributed substantially by co-creating parts of the analysis. An evaluation workshop revealed considerations about time and money investments that must be acknowledged. Particularly, the amount of tacit ‘work’ that PPI requires needs attention in order to balance the investment and the gains. Ethical discussions about the appropriate extent of involvement and potential harms to patients are also important considerations for future studies involving PPI. A small advisory board and patients with some level of methodological competencies in research contributed to a productive and constructive advisory board that shared sincere experiences. This case-study is somewhat unique, in that it had a high degree of workable details, sustainability, and transparency. Moreover, the co-creation through several phases of a clinical study provides an example of ways to incorporate patients’ perspectives in research. This knowledge and experience can be applied to other settings and studies where patients are partners in the research process.

## Supplementary information

**Additional file 1.** GRIPP2 checklist.

## Data Availability

All data and materials supporting the results reported in the article are located at author PCS and can be by requested in Danish. The audio-recorded consultations are not possible to share because individual privacy of patients will then be compromised.

## Abbreviations

PPI: Patient and public involvement
PRO: Patient-reported outcome
VR-CoDES: Verona Coding Definitions of Emotional Sequences
PCORI: Patient-Centered Outcomes Research Institute
PiiAF: Public Involvement Impact Assessment Framework
GRIPP: Guidance for Reporting Involvement of Patients and the Public
PRP: Patient research partner
PI: Principal investigator
